# Punishment of Quacks in Olden Times

**Published:** 1845-03

**Authors:** 


*Punishment of Quacks in Olden Times.*—At a period when quackery is prevailing to such an unprecedented extent amongst us, it may be of advantage to indicate some of the punishments that were formerly inflicted in England on these impudent impostors. We copy the following instances which we find recorded in a recent number of the *Provincial Medical and Surgical Journal:*

"In Edward the Sixth's reign, one Grig, a poulterer, in Surrey, was set in the pillory at Croydon, and again in the Borough of Southwark, during the time of the fair, for cheating people out of their money, by pretending to cure them by charms, by only looking at the patient, or by casting his water.

"In the reign of the first James, the council despatched a warrant to the magistrates of the city of London, to take up all reputed empirics, and bring them before the censors of the college, to examine how properly qualified they were to be trusted either with the limbs or lives of his Majesty's subjects.

"Dr. Lamb, a most noted quack, and who had got a large fortune by his pretended medicines, was at last obliged to confess he knew nothing of physic.

"Read and Woodhouse, two other contemporary quacks, were likewise brought to justice, and acknowledged the same.

"In Stowe's Chronicle we meet with a relation of a water-caster being set on horseback, his face to the horse's tail, which he held in his hand, with a collar of urinals about his neck, led by the hangman through the city, whipped, branded, and then banished.

"Fairfax was fined and imprisoned in King William's time for doing great damage to several persons by his 'Aqua Celestis;' also one Anthony, with his 'Aurum Potabile;' Arthur Dee, for advertising medicines which he gave out would cure people of all diseases; Foster for selling a powder for the green sickness; Aires for selling purging sugar-plums; and Tenant, a urine-caster, who sold his pills at a pound each. Hunt was punished for putting up bills in the streets for the cure of diseases; and Philips, a distiller, for selling strong waters, inserting in the directions what they were good for, and how persons were to take them."

*New York Medical Journal.*

